# Supplementary material for: Realization of active metamaterials with odd micropolar elasticity
Source: Nat Commun. 2021 Oct 12;12:5935. doi: 10.1038/s41467-021-26034-z (PMC8511045; doi:10.1038/s41467-021-26034-z)
Supplement: Supplementary file 1 — SUPPLEMENTARY INFORMATION [file 41467_2021_26034_MOESM1_ESM.pdf]

Supplementary materials for
Realization of active metamaterials with odd micropolar elasticity

Yangyang Chen,[1,*] Xiaopeng Li,[1,*] Colin Scheibner,[2,3,*] Vincenzo Vitelli,[2,3,4,†] and Guoliang Huang[1,‡]

[1] *Department of Mechanical and Aerospace Engineering, University of Missouri, Columbia, MO, 65211, USA*
[2] *James Franck Institute, The University of Chicago, Chicago, IL, 60637, USA*
[3] *Department of Physics, The University of Chicago, Chicago, IL, 60637, USA*
[4] *Kadanoff Center for Theoretical Physics, The University of Chicago, Chicago, IL, 60637, USA*

Contents

Supplementary Note 1: Continuum theory 2
A. Numerical characterization of elastic moduli 2
B. Notions of reciprocity 3
C. Dispersion relations calculated using the discrete model 6
D. Boundary conditions in the continuum 6
E. Boundary conditions in discrete models 9
F. Explicit calculation of eigenmodes 11
G. Role of additional vibrational modes 12
H. Simulations of quasistatic energy cycle and finite frequency efficiency 12

Supplementary Note 2: Experimental details 13
A. Material parameters 13
B. Electrical control system 14

Supplementary Note 3: Numerics 15
A. Transfer matrix method for wave dispersion 15

Supplementary References 19

*These authors contributed equally to this work.
†Electronic address: vitelli@uchicago.edu
‡Electronic address: huangg@missouri.edu

Supplementary Note 1: Continuum theory

A. Numerical characterization of elastic moduli

Our characterization of the elatic moduli begins with the standard kinematic assumptions of Timoshenko beam theory [1]. Let $u_x(x, y, z)$, $u_y(x, y, z)$, and $u_z(x, y, z)$ be the underlying continuum displacement field. Here, the x direction is oriented along the beam, the y direction is oriented out of plane, and the z direction is oriented vertically. In following Timoshenko beam theory, we assume:

1. There are no out-of-plane displacements: $u_y(x, y, z) = 0$.

2. Cross sections of the beam remain planar. Hence, we may introduce the following parameterization:

$$u_z(x, y, 0) = h(x) \tag{S1}$$

$$u_x(x, y, z) = -z\varphi(x) + u(x), \tag{S2}$$

   where $z = 0$ coincides with the midplane of the beam.

Under these assumptions, $h(x)$, $\varphi(x)$, and $u(x)$ are the independent degrees of freedom in our effective 1D model. In the main text, we assume that $u(x) = 0$, as is valid in experiments. Nonetheless, we can probe elongations of the beam in simulation, and hence we include $u(x)$ in our analysis here. The equations of motion are obtained by invoking conservation of angular momentum in the $\hat{y}$ direction, linear momentum in the $\hat{x}$ direction, and linear momentum in the $\hat{z}$ direction. These conserved quantities give rise to the following equations of motion:

$$I\ddot{\varphi} = \partial_x M + \sigma_{zx} \tag{S3}$$

$$\rho\ddot{h} = \partial_x \sigma_{zx} \tag{S4}$$

$$\rho\ddot{u} = \partial_x \sigma_{xx}, \tag{S5}$$

where ρ is the volumetric mass density, I is the cross sectional moment of inertia, M is the bending moment, and σ_{ij} is the stress tensor. Next, we expand the moments and stresses to linear order in the the deformations of the beam, which include bend $\partial_x\varphi$, shear $\partial_x h - \varphi$, and elongation $\partial_x u$. We can summarize the linear response by a 3 by 3 matrix C_{ij}:

$$\begin{pmatrix} \sigma_{zx} \\ M \\ \sigma_{xx} \end{pmatrix} = \begin{pmatrix} C_{11} & C_{12} & C_{13} \\ C_{21} & C_{22} & C_{23} \\ C_{31} & C_{32} & C_{33} \end{pmatrix} \begin{pmatrix} \partial_x h - \varphi \\ \partial_x \varphi \\ \partial_x u \end{pmatrix}. \tag{S6}$$

Eq. (S6) is an enlarged version of Eq. (10) in the main text. It is useful first to make predictions on which moduli will dominate Eq. (S6) based on the properties of the microscopic unit cell presented in Fig. 1 of the main text. First, we expect the beam to inherit the Young's modulus, E, shear modulus μ, and bending moment B present in standard beam theory. Moreover, since elongation or compression of the central piezoelectric sensor induces antisymmetric stresses in the piezoelectric actuator, we expect elongation $\partial_x u$ and bending $\partial_x\varphi$ to give rise to shear stress σ_{zx}. Hence, we expect C_{ij} to take the form:

$$C_{ij} = \begin{pmatrix} \mu & P & K \\ 0 & B & 0 \\ 0 & 0 & E \end{pmatrix} \tag{S7}$$

In Eq. (S7), the moduli K and P are introduced via the piezoelectric feedback. The modulus P is the odd micropolar modulus, which is the primary focus of this work. The modulus K is an asymmetric modulus between shear and elongation.

Supplementary Figure 1 shows the result of finite-element simulations that directly probe the response of the metabeam. The moduli are determined by applying strain controlled boundary conditions at the terminating faces of a single unit cell and computing the reaction forces. We find that $E = 152.4 \times 10^9 \text{kg/ms}^2$, $\mu = 1.3 \times 10^9 \text{kg/ms}^2$, and $B = 0.112 \times 10^6 \text{kg/s}^2$ are independent of the transfer function $H(\omega)$. Moreover, we confirm that when the electronic feedback is present, the linear response $\mathbf{C}$ contains nonzero K and P. We empirically find that K and P are proportional to the transfer function $H(\omega)$ via the relationship $K = \Lambda H$ and $P = \Pi H$, for (real) material constants $\Lambda = 1.2 \times 10^9 \text{kg/ms}^2$ and $\Pi = 4.7 \times 10^6 \text{kgm/s}^2$. Additionally, we find small, but non-zero, values for C_{23} and C_{32}. This coupling between elongation and bending arises since the piezoelectrics are mounted only on the top surface of the metabeam. However, since $|C_{32}|$ and $|C_{23}|$ are small, we can safely neglect these terms in the subsequent analysis.

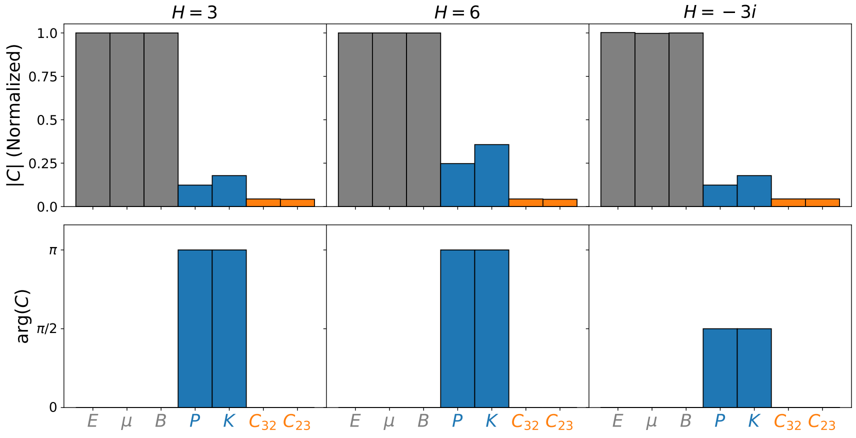

Supplementary Figure 1: Determination of linear response via finite-element simulation. The normalized magnitude and complex argument of the nonzero moduli are shown for three different values of the transfer function H. The quantities E, μ, and B are normalized by their passive values E_0, μ_0, B_0 (determined when $H = 0$). The modulus K is normalized by μ_0. The moduli P, C_{23}, and C_{32} are normalized by $\mu_0 h_b$, where h_b is the thickness of the metabeam (see section Supplementary Note 0 H).

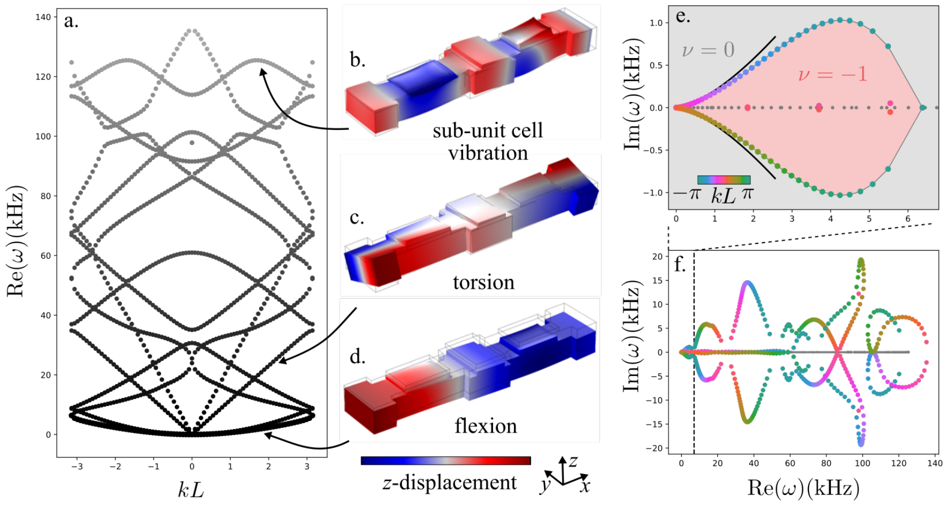

Supplementary Figure 2: **(a)** The fifteen lowest eigenfrequencies computed in COMSOL for the active metabeam with $H(\omega) = 3$, and hence $P = 3\Pi$. **(b-d)** The eigenmodes associated with selected branches. The color indicates the vertical displacement. **(e)** The vibrational spectrum with periodic boundary conditions for low frequencies ($\lesssim$7kHz) are plotted in the complex ω plane. The color of the data points indicates the wave number kL, where L is the length of a unit cell. The solid black lines indicate the continuum theory. For simplicity, only the vibrational frequencies with positive real part are shown. The red region has a winding number of $\nu = -1$ and the grey region has a winding number of $\nu = 0$, as determined by Eq. (30). Numerical modes with negligible imaginary part are represented by the small grey dots. For these simulations, we set the transfer function to $H(\omega) = 3$ and hence $P = 3\Pi$. **(f)** The spectrum for the lowest 15 modes in the complex ω, illustrating the complexity of the full spectrum of the metabeam. Same color scheme as in panel (a).

B. Notions of reciprocity

Here we provide a list of four minimal systems that illustrate the distinction between reciprocity in the sense of momentum conservation and Maxwell-Betti reciprocity. For simplicity, we consider one-dimensional systems for which linear momentum is the relevant form of momentum.

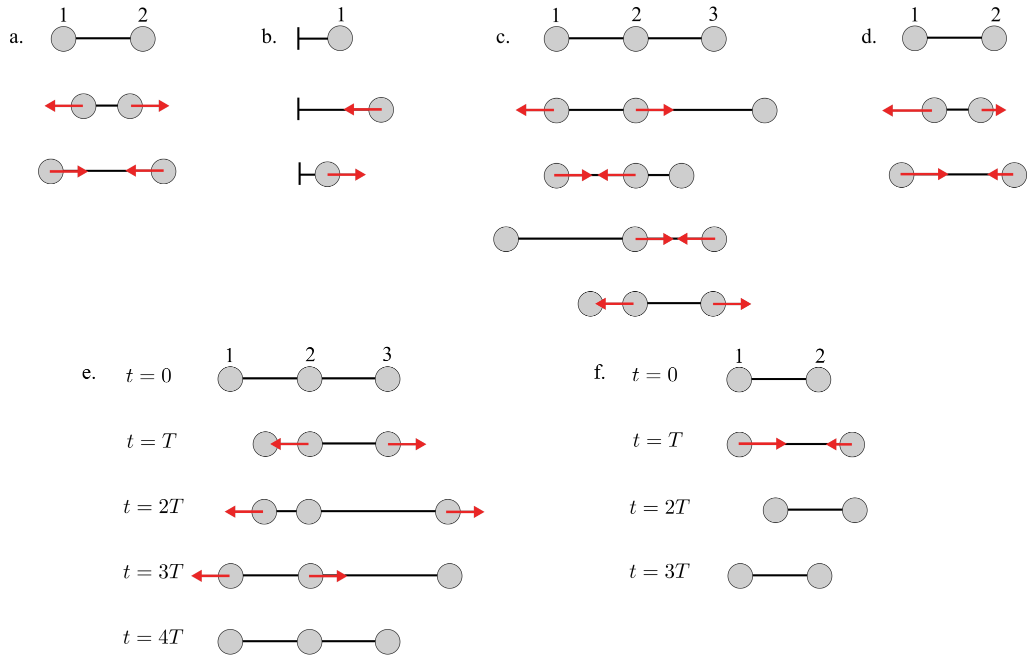

Supplementary Figure 3: **Illustrations of reciprocity violation.** Four model systems are shown in which masses are connected by Hookean springs, or generalizations of Hookean springs. The top row shows the rest state of each system, while deformed states are shown beneath. The red arrows indicate forces. **a.** A Hookean spring exerts equal and opposite forces on the two attached particles when stretched or compressed. This system respects linear momentum conservation and Maxwell-Betti reciprocity. **b.** A mass is connected to a fixed wall by a Hookean spring. Due to the presence of the wall, the linear momentum of the mobile particle is not conserved. Nonetheless, Maxwell-Betti reciprocity is respected. **c.** Three masses are connected by generalized springs. When the right bond (connecting particles 2 and 3) is stretched/compressed, an outward/inward tension is exerted on the left bond (connecting particles 1 and 2). However, when the left bond is stretched/compressed, an inward/outward tension is exerted on the right bond. In each deformed state, the forces on all particles sum to zero, indicating that linear momentum is conserved. Nonetheless, the system violates Maxwell-Betti reciprocity due to the asymmetry in the relationship between the right and left bond. Such a system can be realized via sensors and linear actuators coupled by electronic feedback. Such a system requires an energy source, but no external medium to provide the linear momentum. **d.** A generalized spring is constructed in which the forces acting on the two masses are not equal and opposite. Such a device requires an external source of linear momentum (not shown) to provide the force imbalance, and a source of energy to compensate for the violation of Maxwell-Betti reciprocity. **e-f.** Energy extracting cycles performed with the systems in panels c. and d., respectively.

System 1 The first system is a simple Hookean spring connecting two masses, see Fig. 3a. This system respects both linear momentum conservation and Maxwell-Betti reciprocity. The system has two degrees of freedom u_1 and u_2 being the displacements of the two particles. There are two conjugate forces F_1 and F_2 that are defined via stipulation of the virtual work:

$$\mathrm{d}W = F_1 \mathrm{d}u_1 + F_2 \mathrm{d}u_2 \tag{S8}$$

The system comes with a linear constitutive relation:

$$\begin{bmatrix} F_1 \\ F_2 \end{bmatrix} = \underbrace{\begin{bmatrix} -k & k \\ k & -k \end{bmatrix}}_{\mathbf{C}} \begin{bmatrix} u_1 \\ u_2 \end{bmatrix} \tag{S9}$$

where k is the spring constant. Since the linear response matrix $\mathbf{C}$ is symmetric, the linear response is compatible with a quadratic potential energy $V = \frac{k}{2}(u_1 - u_2)^2$ and hence respects Maxwell-Betti reciprocity. Secondly, we assume dynamic forms $\frac{\mathrm{d}p_1}{\mathrm{d}t} = F_1$ and $\frac{\mathrm{d}p_2}{\mathrm{d}t} = F_2$ where p_1 and p_2 are the momenta associated with masses 1 and 2. The rate of change of the total momentum $P = p_1 + p_2$ is given by:

$$\frac{\mathrm{d}P}{\mathrm{d}t} = \frac{\mathrm{d}p_1}{\mathrm{d}t} + \frac{\mathrm{d}p_2}{\mathrm{d}t} = F_1 + F_2 = 0 \tag{S10}$$

where we have used Eq. (S9).

System 2 The second system we present respects Maxwell-Betti reciprocity, but does not conserve linear momentum. This system is a mass pinned to a substrate by a Hookean spring, see Fig. 3b. This system is described by a single degree of freedom u_1. There is one conjugate force $\mathrm{d}W = F_1 \mathrm{d}u_1$ that is given by

$$F_1 = ku_1 \tag{S11}$$

In this case, the linear response matrix $\mathbf{C}$ is simply a scalar $\mathbf{C} = k$. Thus we have $\mathbf{C} = \mathbf{C}^T$ and the system obeys Maxwell-Betti reciprocity and it is compatible with an energy function $V = \frac{1}{2}u_1^2$. However, the total linear momentum in the system $P = p_1$ is manifestly not conserved since there are configurations of the system for which $\frac{\mathrm{d}P}{\mathrm{d}t} = F_1$ is nonzero. Indeed, linear momentum is conserved if the substrate is included in the analysis. However, we take the point of view that the substrate is not dynamical (since perhaps it is very large) and hence a more reasonable choice of system is the single mass.

System 3 Now we provide an example of a system in which linear momentum is conserved but Maxwell-Betti reciprocity is violated. As shown in Fig. 3c, consider a collection of three coupled masses with three degrees of freedom u_1, u_2, and u_3. The the system has three conjugate forces defined through a statement of virtual work $\mathrm{d}W = F_1\mathrm{d}u_1 + F_2\mathrm{d}u_2 + F_3\mathrm{d}u_3$. The masses are coupled through specially designed actuators such that they have the following constitutive relationship

$$\begin{bmatrix} F_1 \\ F_2 \\ F_3 \end{bmatrix} = \underbrace{\begin{bmatrix} 0 & k^a & -k^a \\ -k^a & 0 & k^a \\ k^a & -k^a & 0 \end{bmatrix}}_{\mathbf{C}} \begin{bmatrix} u_1 \\ u_2 \\ u_3 \end{bmatrix} \tag{S12}$$

In this case, the linear response matrix $\mathbf{C}$ is not symmetric. This is an indication that the system violates Maxwell-Betti reciprocity. To see that an energy function cannot be defined, consider the following protocol illustrated in Fig. 3e.

$$u_1(t) = \begin{cases} tU & t \in [0, T] \\ TU & t \in [T, 2T] \\ U(3T - t) & t \in [2T, 3T] \\ 0 & t \in [3T, 4T]. \end{cases} \tag{S13}$$

$$u_2(t) = 0 \tag{S14}$$

$$u_3(t) = \begin{cases} 0 & t \in [0, T] \\ (t - T)U & t \in [T, 2T] \\ UT & t \in [2T, 3T] \\ U(4T - t) & t \in [3T, 4T]. \end{cases} \tag{S15}$$

The work done along this closed cycle in the configuration space can be easily computed to be $\int_0^{4T} \mathrm{d}W = \frac{1}{2}U^2k^a$. Since the work is nonzero along a closed cycle, a potential energy function cannot be defined. Nonetheless, the total linear momentum $P = p_1 + p_2 + p_3$ is conserved since:

$$\frac{\mathrm{d}P}{\mathrm{d}t} = F_1 + F_2 + F_3 = 0 \tag{S16}$$

for all configurations.

System 4 Finally, we consider an example that violates both Maxwell-Betti reciprocity and linear momentum conservation [2]. The model has two degrees of freedom u_1 and u_2 and two conjugate forces F_1 and F_2. The constitutive relation is given by:

$$\begin{bmatrix} F_1 \\ F_2 \end{bmatrix} = \underbrace{\begin{bmatrix} -k(1+\epsilon) & k(1+\epsilon) \\ k(1-\epsilon) & -k(1-\epsilon) \end{bmatrix}}_{\mathbf{C}} \begin{bmatrix} u_1 \\ u_2 \end{bmatrix} \tag{S17}$$

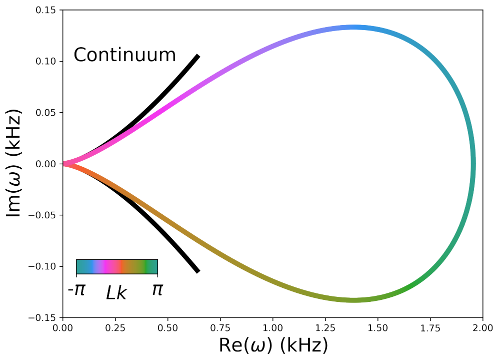

Supplementary Figure 4: **Dispersion relations of the discrete model of the odd micropolar metabeam calculated**. Here we use for $p > 0$ (parameters are the same as those in Fig. 3a), and the color bar represents the normalized wavenumber kL. See Section IIF of the main text for the description of the model. The solid black line represents the continuum theory with wave number $kL \in [-0.96, 0.96]$.

Here, the linear response matrix is no longer symmetric $\mathbf{C} \neq \mathbf{C}^T$, indicating that Maxwell-Betti reciprocity is violated. Moreover, as illustrated in Fig. 3f, one can perform a cycle in the configuration space that extracts work:

$$u_1(t) = \begin{cases} 0 & t \in [0, T] \\ (t-T)U & t \in [T, 2T] \\ UT & t \in [2T, 3T] \end{cases} \tag{S18}$$

$$u_2(t) = \begin{cases} Ut & t \in [0, T] \\ TU & t \in [T, 2T] \\ U(3T-t) & t \in [2T, 3T] \end{cases} \tag{S19}$$

The total work done is $\int_0^{3T} \mathrm{d}W = 2k\epsilon U^2$. However, the total linear momentum $P = p_1 + p_2$ is not conserved since

$$\frac{\mathrm{d}P}{\mathrm{d}t} = -k\epsilon(u_1 - u_2) \tag{S20}$$

which is nonvanishing. To physically realize such a system, a linear momentum sink must be present in addition to a source of energy to provide the Maxwell-Betti reciprocity violation.

C. Dispersion relations calculated using the discrete model

Fig. S4 shows a comparison of the dispersion relations for the discrete model and continuum theory.

D. Boundary conditions in the continuum

Here we derive the index Eqs. (M6) and (M7) in the main text. For concreteness, we will perform the derivation in the context of the continuum theory for the active metabeam, with sufficient discussion to allow generalization. For further discussion, see Ref. [3] and references therein. Consider a system with semi-infinite boundary conditions $[0, \infty)$ governed by the dynamical matrix $\boldsymbol{D}(k)$. In the notation of Eq. (23), we seek to determine many vectors $\boldsymbol{\Psi} = (\tilde{p}_h, \tilde{p}_\varphi \tilde{s}, \tilde{b})$ satisfy the equation

$$[\boldsymbol{D}(k) - \omega] \cdot \boldsymbol{\Psi} = 0 \tag{S21}$$

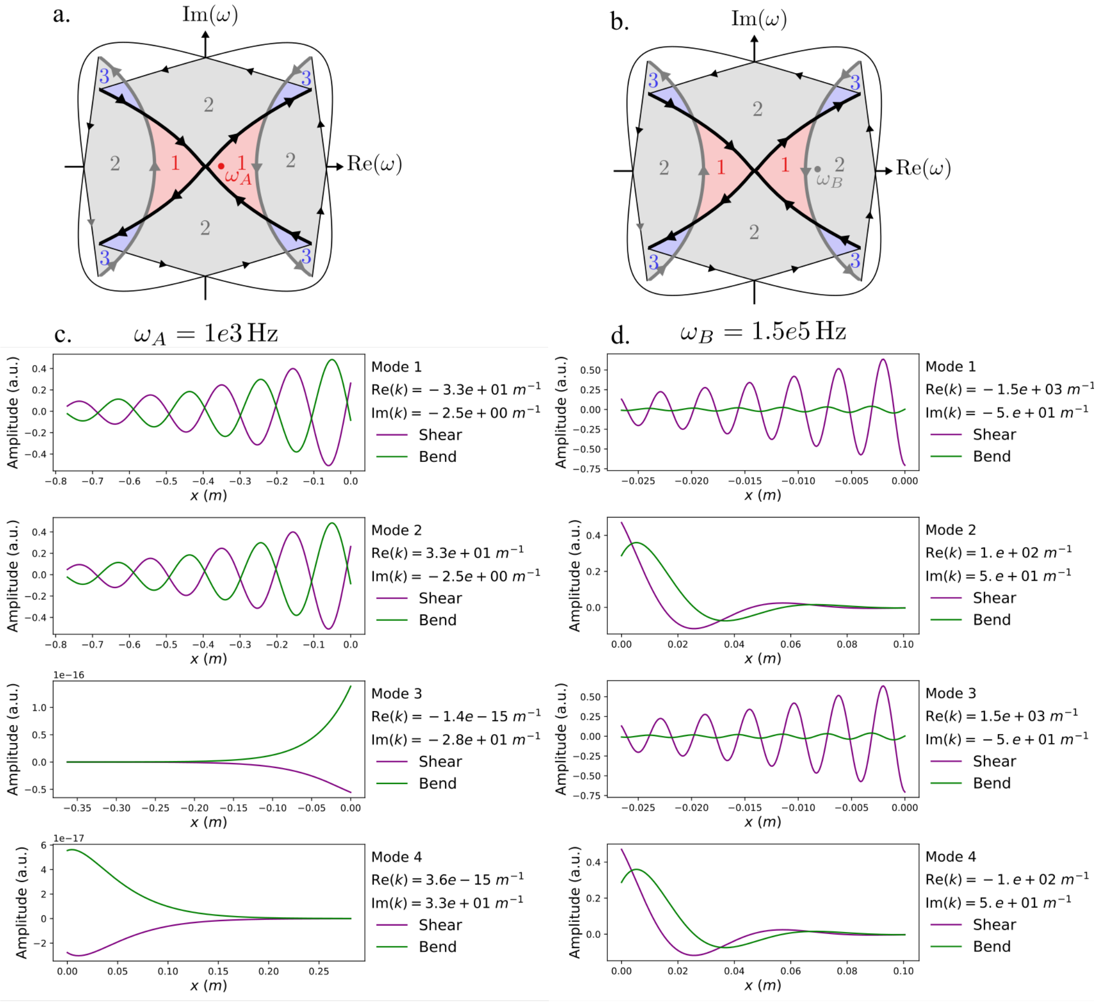

Supplementary Figure 5: **Illustration of localized modes from continuum theory. a-b.** The spectrum in the complex plane for $P > 0$, using notation identical to that used in Figure M1. Here we highlight two modes $\omega_A = 1 \times 10^3$Hz and $\omega_B = 1.5 \times 10^5$Hz, schematically placed within the complex plane. We compute the eigenmodes of Eq. (S44) at frequencies ω_A and ω_B using the parameter $\omega_1 = 10^5$Hz, $\ell_1 = 10^{-3}m$, $\ell_2 = 10^{-2}m$, $\tilde{P} = 1$. In **c-d.** we show the shearing $\tilde{s}$ and bending $\tilde{b}$ profiles over space. Consistent with the winding number $\tilde{\nu}(\omega_A) = 1$, there is one left localized mode and three right localized modes at ω_A. Moreover, we have $\tilde{\nu}(\omega_B) = 2$, which is consistent with two left-localized modes and two right-localized modes.

with $\mathrm{Im}(k) > 0$ subject to γ *independent*, *homogeneous* boundary conditions at $x = 0$. By independent, homogeneous boundary conditions, we mean boundary conditions that can be stated in the following form:

$$\begin{bmatrix} B_{1,1} & \dots & B_{1,4(N+1)} \\ \vdots & & \vdots \\ B_{\gamma,1} & \dots & B_{\gamma,4(N+1)} \end{bmatrix} \begin{bmatrix} \tilde{p}_h(x) \\ \tilde{p}_\varphi(x) \\ \tilde{s}(x) \\ \tilde{b}(x) \\ \vdots \\ \partial_x^N \tilde{p}_h(x) \\ \partial_x^N \tilde{p}_\varphi(x) \\ \partial_x^N \tilde{s}(x) \\ \partial_x^N \tilde{b}(x) \end{bmatrix}_{x=0} = 0 \tag{S22}$$

where $\mathbf{B}$ is a matrix of rank γ. To count the number of solutions to $[\boldsymbol{D}(k)-\omega]\cdot\boldsymbol{\Psi}=0$, we introduce the winding number

$$\tilde{\nu}(\omega)=\lim_{R\to\infty}\frac{1}{2\pi i}\oint_{\Gamma(R)}\frac{\mathrm{d}}{\mathrm{d}k}\log f(k)\mathrm{d}k \tag{S23}$$

where $f(k)=\det[\boldsymbol{D}(k)-\omega]$ and $\Gamma(R)$ is the contour $[-R,R]$ completed by $Re^{i\phi}$ with $\phi\in[0,\pi]$. Notice that $f(k)$ is a polynomial with non-negative powers of k. Let d be the rank of the polynomial $f(k)$. To simplify the derivation below, we will assume without loss of generality that $4N\geq d$. This is not a restriction because it can always be achieved by augmenting $\mathbf{B}$ with zero columns. Since $f(k)$ contains only positive powers of k, the poles of $f(k)$ are located at $|k|\to\infty$ and hence are not included in the contour $\Gamma(R)$ for any R. Thus, by Cauchy's argument principle, $\tilde{\nu}(\omega)$ counts the number of zeros of $f(k)$ with $\mathrm{Im}(k)>0$. Each zero k_a has an associated "candidate" eigenvector Ψ^a, with $a=1,2,\ldots,\tilde{\nu}$. Therefore, we may write:

$$\begin{bmatrix}\tilde{p}_h(x)\\ \tilde{p}_\varphi(x)\\ \tilde{s}(x)\\ \tilde{b}(x)\\ \vdots\\ \partial_x^N\tilde{p}_h(x)\\ \partial_x^N\tilde{p}_\varphi(x)\\ \partial_x^N\tilde{s}(x)\\ \partial_x^N\tilde{b}(x)\end{bmatrix}_{x=0}=\underbrace{\begin{bmatrix}\tilde{p}_h^1 & \cdots & \tilde{p}_h^{\tilde{\nu}}\\ \tilde{p}_\varphi^1 & \cdots & \tilde{p}_\varphi^{\tilde{\nu}}\\ \tilde{s}^1 & \cdots & \tilde{s}^{\tilde{\nu}}\\ \tilde{b}^1 & \cdots & \tilde{b}^{\tilde{\nu}}\\ \vdots & & \\ k_1^N\tilde{p}_h^1 & \cdots & k_{\tilde{\nu}}^N\tilde{p}_h^{\tilde{\nu}}\\ k_1^N\tilde{p}_\varphi^1 & \cdots & k_{\tilde{\nu}}^N\tilde{p}_\varphi^{\tilde{\nu}}\\ k_1^N\tilde{s}^1 & \cdots & k_{\tilde{\nu}}^N\tilde{s}^{\tilde{\nu}}\\ k_1^N\tilde{b}^1 & \cdots & k_{\tilde{\nu}}^N\tilde{b}^{\tilde{\nu}}\end{bmatrix}}_{\mathbf{H}}\begin{bmatrix}g_1\\ g_2\\ \vdots\\ g_{\tilde{\nu}}\end{bmatrix} \tag{S24}$$

We seek to determine the number of linearly independent vectors $\mathbf{g}$ that satisfy the boundary conditions $\mathbf{B}\cdot\mathbf{H}\cdot\mathbf{g}=0$. Thus, we seek to determine $\dim\ker\mathbf{B}\cdot\mathbf{H}$. Since $4N\geq d\geq\tilde{\nu}$, the columns of $\mathbf{H}$ are linearly independent and hence $\mathrm{rank}\,\mathbf{H}=\tilde{\nu}$. Therefore, we may write:

$$\mathbf{B}=\begin{bmatrix}\mathbf{a}_1 & \mathbf{a}_2 & \cdots & \mathbf{a}_\gamma\end{bmatrix}\begin{bmatrix}\mathbf{b}_1^T\\ \mathbf{b}_2^T\\ \vdots\\ \mathbf{b}_\gamma^T\end{bmatrix} \tag{S25}$$

$$\mathbf{H}=\begin{bmatrix}\mathbf{c}_1 & \mathbf{c}_2 & \cdots & \mathbf{c}_{\tilde{\nu}}\end{bmatrix}\begin{bmatrix}\mathbf{d}_1^T\\ \mathbf{d}_2^T\\ \vdots\\ \mathbf{d}_{\tilde{\nu}}^T\end{bmatrix} \tag{S26}$$

(S27)

where each set of vectors $\{\mathbf{a}_i\}$, $\{\mathbf{b}_i\}$, $\{\mathbf{c}_i\}$, $\{\mathbf{d}_i\}$ are individually linearly independent. Therefore, we may write

$$\mathbf{B}\cdot\mathbf{H}=\begin{bmatrix}\mathbf{a}_1 & \mathbf{a}_2 & \cdots & \mathbf{a}_\gamma\end{bmatrix}\underbrace{\begin{bmatrix}\mathbf{b}_1\cdot\mathbf{c}_1 & \mathbf{b}_1\cdot\mathbf{c}_2 & \cdots & \mathbf{b}_1\cdot\mathbf{c}_{\tilde{\nu}}\\ \mathbf{b}_2\cdot\mathbf{c}_1 & \mathbf{b}_2\cdot\mathbf{c}_2 & \cdots & \mathbf{b}_2\cdot\mathbf{c}_{\tilde{\nu}}\\ \vdots & \vdots & \ddots & \vdots\\ \mathbf{b}_\gamma\cdot\mathbf{c}_1 & \mathbf{b}_\gamma\cdot\mathbf{c}_2 & \cdots & \mathbf{b}_\gamma\cdot\mathbf{c}_{\tilde{\nu}}\end{bmatrix}}_{\mathbf{G}}\begin{bmatrix}\mathbf{d}_1^T\\ \mathbf{d}_2^T\\ \vdots\\ \mathbf{d}_{\tilde{\nu}}^T\end{bmatrix} \tag{S28}$$

For any generic $\boldsymbol{D}(k)$ and generic $\omega\in\mathbb{C}$, the singular values of $\mathbf{G}$ will be nonzero. (By *generic*, we mean that the only exceptions, if any, constitute a measure zero sets). In this case, we have $\mathrm{rank}\,\mathbf{B}\cdot\mathbf{H}=\min(\tilde{\nu},\gamma)$. Hence by the rank nullity theorem, $\dim\ker\mathbf{B}\cdot\mathbf{H}=\tilde{\nu}-\min(\tilde{\nu},\gamma)$, which is the index theorem given in the methods. For solutions on the domain $x\in(-\infty,0]$, a similar derivation applies. The difference being that $f(k)$ has $d-\tilde{\nu}(\omega)$ zeros with $\mathrm{Im}(k)<0$.

We now provide two examples physically relevant independent homogeneous boundary conditions. Stress-free boundary conditions can be formulated by choosing $\mathbf{B}$ as follows:

$$\mathbf{B}=\begin{bmatrix}0 & 0 & C_{11} & C_{12}\\ 0 & 0 & C_{21} & C_{22}\end{bmatrix} \tag{S29}$$

where $\mathbf{C}$ is the constitutive matrix. For motion-free boundary conditions, one may write

$$\mathbf{B} = \begin{bmatrix} 1 & 0 & 0 & 0 \\ 0 & 1 & 0 & 0 \end{bmatrix} \tag{S30}$$

which imposes that $\tilde{p}_h = \tilde{p}_\varphi = 0$ at the boundary, i.e. there is no rotation or translation in time at the boundary. To impose displacement free boundary conditions, one can reformulate the linear differential equation such that $\boldsymbol{\Psi} = (\tilde{p}_h, \tilde{p}_\varphi, h, \varphi)$ instead of $\boldsymbol{\Psi} = (\tilde{p}_h, \tilde{p}_\varphi, \tilde{s}, \tilde{b})$.

E. Boundary conditions in discrete models

Here we derive and interpret $\nu(\omega)$ in discrete settings, such as discrete models or finite element simulations. We refer the reader to Refs. [3, 4], and references therein, for additional discussion. For concreteness, we will illustrate each step with the discrete model in Section IIF of the main text. To begin, suppose that the ith unit cell of the discrete model is described by an n-component vector $\boldsymbol{\Psi}_i$. For the example at hand, we have $n = 4$ and $\boldsymbol{\Psi}_i = (p_i, l_i, h_i, \varphi_i)$ where $p_i = m\dot{h}_i$ is the vertical linear momentum and $l_i = J\dot{\varphi}_i$ is the angular momentum of each unit cell about its center of mass. The linear equations of motion for an infinite system take the form:

$$i\partial_t \begin{bmatrix} \vdots \\ \boldsymbol{\Psi}_{i-1} \\ \boldsymbol{\Psi}_i \\ \boldsymbol{\Psi}_{i+1} \\ \vdots \end{bmatrix} = \underbrace{\begin{bmatrix} \ddots & & \ddots & \ddots & \ddots & & \ddots & & \\ & \boldsymbol{\mathcal{D}}_l & \cdots & \boldsymbol{\mathcal{D}}_{-1} & \boldsymbol{\mathcal{D}}_0 & \boldsymbol{\mathcal{D}}_1 & \cdots & \boldsymbol{\mathcal{D}}_r & 0 \\ & 0 & \boldsymbol{\mathcal{D}}_l & \cdots & \boldsymbol{\mathcal{D}}_{-1} & \boldsymbol{\mathcal{D}}_0 & \boldsymbol{\mathcal{D}}_1 & \cdots & \boldsymbol{\mathcal{D}}_r \\ & & \ddots & & \ddots & \ddots & & & \ddots \end{bmatrix}}_{\boldsymbol{\mathcal{D}}} \begin{bmatrix} \vdots \\ \boldsymbol{\Psi}_{i-1} \\ \boldsymbol{\Psi}_i \\ \boldsymbol{\Psi}_{i+1} \\ \vdots \end{bmatrix} \tag{S31}$$

where the matrix $\boldsymbol{\mathcal{D}}$ is an infinite matrix formally known as an *Laurent operator.* Each $n \times n$ matrix $\boldsymbol{\mathcal{D}}_i$ lies in the ith off diagonal, with i ranging from $l < 0$ to $r > 0$. For the system at hand, we have $l = r = -1$, and the equations of motion in Eqs. (21-22) of the main text take the form

$$i\partial_t \begin{bmatrix} \vdots \\ \boldsymbol{\Psi}_{i-1} \\ \boldsymbol{\Psi}_i \\ \boldsymbol{\Psi}_{i+1} \\ \vdots \end{bmatrix} = \begin{bmatrix} \ddots & \ddots & & & \\ \ddots & \boldsymbol{\mathcal{D}}_0 & \boldsymbol{\mathcal{D}}_1 & 0 & \\ & \boldsymbol{\mathcal{D}}_{-1} & \boldsymbol{\mathcal{D}}_0 & \boldsymbol{\mathcal{D}}_1 & \\ & 0 & \boldsymbol{\mathcal{D}}_{-1} & \boldsymbol{\mathcal{D}}_0 & \ddots \\ & & & \ddots & \ddots \end{bmatrix} \begin{bmatrix} \vdots \\ \boldsymbol{\Psi}_{i-1} \\ \boldsymbol{\Psi}_i \\ \boldsymbol{\Psi}_{i+1} \\ \vdots \end{bmatrix} \tag{S32}$$

with

$$\boldsymbol{\mathcal{D}}_0 = i \begin{bmatrix} 0 & 0 & -2k_\mu & -k_\mu L - 2p \\ 0 & 0 & -k_\mu L & -k_\mu L^2 - 2\kappa_B - 2pL \\ \frac{1}{m} & 0 & 0 & 0 \\ 0 & \frac{1}{J} & 0 & 0 \end{bmatrix} \tag{S33}$$

$$\boldsymbol{\mathcal{D}}_1 = i \begin{bmatrix} 0 & 0 & k_\mu & p \\ 0 & 0 & k_\mu L & \kappa_B + pL \\ 0 & 0 & 0 & 0 \\ 0 & 0 & 0 & 0 \end{bmatrix} \tag{S34}$$

$$\boldsymbol{\mathcal{D}}_{-1} = i \begin{bmatrix} 0 & 0 & k_\mu & Lk_\mu + p \\ 0 & 0 & 0 & \kappa_B \\ 0 & 0 & 0 & 0 \end{bmatrix} \tag{S35}$$

For an infinite system, one is interested in the spectrum of $\boldsymbol{\mathcal{D}}$. To find the eigenvalues, one can perform a Fourier transform to obtain the symbol

$$\boldsymbol{\mathcal{D}}(\lambda) = \sum_{i=l}^{r} \boldsymbol{\mathcal{D}}_i \lambda^i \tag{S36}$$

where $\lambda = e^{ikL}$ with k being the wavenumber and L being the lattice spacing. We are then interested in the solutions to the equation:

$$F(\lambda) \equiv \det[\boldsymbol{\mathcal{D}}(\lambda) - \omega] = 0 \tag{S37}$$

For the example at hand,

$$\begin{aligned} F(\lambda) =& \frac{k_\mu \kappa_B}{Jm}\lambda^2 - \frac{4k_\mu\kappa_B - (Jk_\mu + Lmp + m\kappa_B)\omega^2}{Jm}\lambda \\ &+ \frac{2k_\mu Lp + 6k_\mu\kappa_B - (2Jk_\mu + k_\mu L^2 m + 2Lmp + 2m\kappa_B)\omega^2 + Jm\omega^4}{Jm} \\ &- \frac{4k_\mu\kappa_B - (Jk_\mu + m\kappa_B)\omega^2}{Jm}\frac{1}{\lambda} + \frac{k_\mu\kappa_B}{Jm}\frac{1}{\lambda^2} \end{aligned} \tag{S38}$$

For the semi-infinite system bounded on the left, then we are interested in the solutions of the following equation:

$$i\partial_t \begin{bmatrix} \boldsymbol{\Psi}_0 \\ \boldsymbol{\Psi}_1 \\ \boldsymbol{\Psi}_2 \\ \vdots \end{bmatrix} = \underbrace{\begin{bmatrix} \boldsymbol{\mathcal{D}}_0 & \boldsymbol{\mathcal{D}}_1 & \cdots & \boldsymbol{\mathcal{D}}_r & 0 & & & & \\ \boldsymbol{\mathcal{D}}_{-1} & \boldsymbol{\mathcal{D}}_0 & \boldsymbol{\mathcal{D}}_1 & \cdots & \boldsymbol{\mathcal{D}}_r & 0 & & & \\ \vdots & \ddots & \ddots & \ddots & & \ddots & \ddots & & \\ \boldsymbol{\mathcal{D}}_l & \cdots & \boldsymbol{\mathcal{D}}_{-1} & \boldsymbol{\mathcal{D}}_0 & \boldsymbol{\mathcal{D}}_1 & \cdots & \boldsymbol{\mathcal{D}}_r & 0 & \\ 0 & \boldsymbol{\mathcal{D}}_l & \cdots & \boldsymbol{\mathcal{D}}_{-1} & \boldsymbol{\mathcal{D}}_0 & D_1 & \cdots & \boldsymbol{\mathcal{D}}_r & 0 \\ & & \ddots & & \ddots & \ddots & \ddots & & \ddots \end{bmatrix}}_{\tilde{\boldsymbol{\mathcal{D}}}_L} \begin{bmatrix} \boldsymbol{\Psi}_0 \\ \boldsymbol{\Psi}_1 \\ \boldsymbol{\Psi}_2 \\ \vdots \end{bmatrix} \tag{S39}$$

Here $\tilde{\boldsymbol{\mathcal{D}}}_L$ is a semi-infinite matrix known as a *Toeplitz operator*, which is the truncation of a Laurent operator. We seek to determine the number of eigenvectors of $\tilde{\boldsymbol{\mathcal{D}}}_L$ with eigenvalue ω, or equivalently $\dim\ker[\tilde{\boldsymbol{\mathcal{D}}}_L - \omega]$. Notice that $\ker[\tilde{\boldsymbol{\mathcal{D}}}_L - \omega]$ is isomporphic to the subspace of $\ker[\boldsymbol{\mathcal{D}} - \omega]$ that obeys the constraint:

$$0 = \underbrace{\begin{bmatrix} \boldsymbol{\mathcal{D}}_{-1} & 0 & 0 & 0 \\ \boldsymbol{\mathcal{D}}_{-2} & \boldsymbol{\mathcal{D}}_{-1} & 0 & 0 \\ \vdots & \ddots & \ddots & 0 \\ \boldsymbol{\mathcal{D}}_l & \cdots & \boldsymbol{\mathcal{D}}_{-2} & \boldsymbol{\mathcal{D}}_{-1} \end{bmatrix}}_{\mathbf{M}_L} \begin{bmatrix} \Psi_l \\ \Psi_{l+1} \\ \vdots \\ \Psi_{-1} \end{bmatrix} \tag{S40}$$

The constraint in Eq. (S40) can be interpreted as the boundary conditions implied by the truncation of the Laurent operator. The number of independent boundary conditions is given by $\gamma_L = \operatorname{rank}\mathbf{M}_L$. For the discrete model of the beam, the constraint reads $\boldsymbol{\mathcal{D}}_{-1}\cdot\boldsymbol{\Psi}_{-1} = 0$, or equivalently $h_{-1} = 0$ and $\varphi_{-1} = 0$, which are displacement and rotation free boundaries. Notice that in general, the form of $\mathbf{M}_L$ depends on how the continuum equation is discretized. More generally, one can show that $\operatorname{rank}\mathbf{M} = \deg F(1/\lambda)$, where deg denotes the highest power appearing in the polynomial. For the discrete model of the odd micropolar beam, we have $\gamma_L = 2$.

Next we consider the integral

$$\nu(\omega) = \frac{1}{2\pi i}\oint_{S^1} \frac{\mathrm{d}}{\mathrm{d}\lambda}\log F(\lambda)\mathrm{d}\lambda \tag{S41}$$

where S^1 is the unit circle. By Cauchy's argument principle, $\nu(\omega)$ counts with multiplicity the number of zeros of minus the number of poles of $F(\lambda)$ with $|\lambda| \leq 1$ (or equivalently $\mathrm{Im}(k) > 0$). Each of the zeros represents and eigenmode of $\boldsymbol{\mathcal{D}}(\lambda)$ there are exactly γ_L poles at $\lambda = 0$ corresponding to the γ_L constraints imposed by the matrix $\mathbf{M}$. Next, supposing $\nu + \gamma_L > 0$, let λ_a and $\boldsymbol{\Psi}_i^a$, with $a = 1, 2, \ldots, \nu(\omega) + \gamma_L$ be the roots and eigenvectors, respectively. Then we may define the matrix

$$H = \begin{bmatrix} \boldsymbol{\Psi}_l^1 & \cdots & \boldsymbol{\Psi}_l^{\nu+\gamma_L} \\ \vdots & & \vdots \\ \boldsymbol{\Psi}_{-1}^1 & \cdots & \boldsymbol{\Psi}_{-1}^{\nu+\gamma_L} \end{bmatrix} \tag{S42}$$

We seek to determine $\dim\ker \mathbf{M}_L \cdot \mathbf{H}$. Notice that $\operatorname{rank}\mathbf{H} = \min(\nu + \gamma_L, n|l|)$ and $\operatorname{rank}\mathbf{M} = \gamma_L < n|l|$. Therefore, for a generic $\boldsymbol{\mathcal{D}}(\lambda)$ and a generic $\omega \in \mathbb{C}$ we have $\operatorname{rank}\mathbf{M}_L \cdot \mathbf{H} = \min(\gamma_L, \nu + \gamma_L)$. Hence, by the rank-nullity theorem, $\dim\ker \mathbf{M}_L \cdot \mathbf{H} = \max(0, \nu)$. Thus there will generically be $\max[\nu(\omega), 0]$ eigenmodes of $\tilde{\boldsymbol{\mathcal{D}}}_L$ at frequency ω localized to the left boundary. Likewise, to study eigenmodes localized to the right, one obtains $\tilde{\boldsymbol{\mathcal{D}}}_R$ by truncating $\boldsymbol{\mathcal{D}}$ in the opposite direction. In this case, one is interested in the constraint matrix:

$$\mathbf{M}_R = \begin{bmatrix} \boldsymbol{\mathcal{D}}_l & \boldsymbol{\mathcal{D}}_2 & \cdots & \boldsymbol{\mathcal{D}}_1 \\ \vdots & & \iddots & \\ \boldsymbol{\mathcal{D}}_2 & \boldsymbol{\mathcal{D}}_1 & & \\ \boldsymbol{\mathcal{D}}_1 & & & \end{bmatrix} \tag{S43}$$

For $\mathbf{M}_R$, we have $\gamma_L = \operatorname{rank}\mathbf{M}_R = \deg F(\lambda)$. Repeating the argument above, for a generic $\boldsymbol{\mathcal{D}}(\lambda)$ and $\omega \in \mathbb{C}$, one has $\operatorname{rank}\ker \mathbf{M}_R \cdot \mathbf{H} = \max[0, -\nu(\omega)]$ and thus there will be $\max[0, -\nu(\omega)]$ eigenmodes of $\tilde{\boldsymbol{\mathcal{D}}}_R$ localized to the right boundary.

Finally, we discuss the application to numerical eigenmode solvers. The COMSOL simulations impose a finite element mesh on a single unit cell, whose points form the content of the vector $\boldsymbol{\Psi}_i$ that is governed by an $n \times n$ matrix $\boldsymbol{\mathcal{D}}(\lambda)$, now with a much larger n. When solving for eigenmodes at a given wavenumber k, COMSOL requires that the state of the right boundary is equal to the state of the left boundary of the mesh multiplied by a phase e^{ikL}. This is equivalent to implementing surface coupling between nearest neighbor unit cells, corresponding to $r = -l = 1$. For this surface coupling, the constraint matrices $\mathbf{M}_R$ and $\mathbf{M}_L$ effectively impose motion-free boundary conditions. In the continuum, motion-free boundaries are captured by Eq. (S30), for which $\gamma = 2$. Hence, for the data in Fig. 3 and Fig. M1, one should compare $\nu(\omega) = \tilde{\nu}(\omega) - 2$.

F. Explicit calculation of eigenmodes

Here we provide explicit calculations of eigenmodes in the continuum theory. The starting place is the wave equation in Eq. (23) of the main text, repeated here:

$$\omega \begin{bmatrix} \tilde{p}_h \\ \tilde{p}_\varphi \\ \tilde{s} \\ \tilde{b} \end{bmatrix} = \omega_1 \underbrace{\begin{bmatrix} 0 & 0 & -k\ell_1 & -\tilde{P}\ell_1 k \\ 0 & 0 & i & i\tilde{P} - k\ell_2 \\ -k\ell_1 & -i & 0 & 0 \\ 0 & -k\ell_2 & 0 & 0 \end{bmatrix}}_{\boldsymbol{D}(k)} \begin{bmatrix} \tilde{p}_h \\ \tilde{p}_\varphi \\ \tilde{s} \\ \tilde{b} \end{bmatrix} \tag{S44}$$

The secular equation is given by

$$0 = \tilde{\omega}^4 - \Big[1 - i\tilde{P}k\ell_2 + k^2(\ell_1^2 + \ell_2^2)\Big]\tilde{\omega}^2 + k^4\ell_1^2\ell_2^2 \tag{S45}$$

First notice that when $P = 0$ and to leading order in $k = 0$, the eigensystem of $\boldsymbol{D}(k)$ takes the following form:

$$\begin{bmatrix} \tilde{p}_h \\ \tilde{p}_\varphi \\ \tilde{s} \\ \tilde{b} \end{bmatrix} = \begin{bmatrix} \pm i \\ k\ell_1 \\ \pm ik\ell_2 \\ 1 \end{bmatrix} \quad \text{with } \omega = \pm k^2 \ell_1 \ell_2 \omega_1 \tag{S46}$$

which represents a bending dominated goldstone mode. The second two modes are shearing dominated gaped modes:

$$\begin{bmatrix} \tilde{p}_h \\ \tilde{p}_\varphi \\ \tilde{s} \\ \tilde{b} \end{bmatrix} = \begin{bmatrix} \pm ik\ell_1 \\ \pm 1 \\ i \\ k\ell_2 \end{bmatrix} \quad \text{with } \omega = \pm\left(1 - k^2 \frac{\ell_1^2 + \ell_2^2}{2}\right)\omega_1 \tag{S47}$$

When $P > 0$, we proceed numerically. Let us use numerical values representative of the experiment, $\ell_1 = 10^{-3}$m, $\ell_2 = 10^{-2}$m, $\tilde{P} = 1$, and $\omega_1 = 10^5$Hz. We will consider the modes at two select frequencies: $\omega_A = 10^3$Hz and $\omega_B = 10^6$Hz. Notice that $\omega_A \ll \omega_1 \ll \omega_B$ and that the degree of Eq. (S45), treated as a polynomial in k, is $d = 4$. From the location of ω_A and ω_B in the complex plane, we have $\nu(\omega_A) = 1$ and $\nu(\omega_B) = 2$, see Fig. 5a-b. Therefore,

for ω_A, we expect one solution to Eq. (S45) left-localized solution ($\mathrm{Im}(k) > 0$) and $d - \nu(\omega_A) = 3$ right-localized solutions ($\mathrm{Im}(k) < 0$), see Fig. 5c-d. We plot the corresponding eigenmode of $D(k)$ for each of these values, and we find that there is stronger mixing between bending and shearing than in the passive case.

To illustrate the meaning of $\tilde{\nu}(\omega_A) = 1$, suppose we impose two boundary conditions ($\gamma = 2$) such as $\tilde{s} = \tilde{b} = 0$ at $x = 0$. Given that there is only one left-localized mode, the two boundary conditions cannot be satisfied for a system with a boundary on the left. However, there are three right-localized modes so there is one nonzero linear combination of right localized modes that satisfy the boundary conditions. We can repeat the calculation now for ω_B, for which we have $\tilde{\nu}(\omega_B) = 2$. Hence we expect $\tilde{\nu}(\omega_B) = 2$ left localized modes and $d - \tilde{\nu}(\omega_B) = 2$ right localized modes before considering boundary conditions. Once we impose $\gamma = 2$ boundary conditions (e.g. $\tilde{s} = \tilde{b} = 0$), one finds that it is not possible to formulate a linear combination for a nonzero mode at either boundary.

G. Role of additional vibrational modes

As shown in Fig. M1 of the main text, the continuum theory predicts a total of four vibrational modes: two flexural goldstone modes and two gapped, shear dominated modes. However, at high frequencies, comparable to ω_1, the continuum theory is not expected to self-consistently apply. Hence, the shear dominated modes should not necessarily be thought of as a physical prediction made by the continuum theory. Moreover, there is a second simplifications invoked when using this continuum theory. The physical beam has a total of four goldstone modes: a flexural mode, a torsional mode, an out-of-plane deformation, and a longitudinal mode. Our ability to apply the continuum theory to the physical beam rests on two prerequisites. First the experiments probe frequencies on the order of $\lesssim$ 10kHz, well below $\omega_1 \approx$ 100kHz. Second, based on the construction of the piezoelectric feedback, we assume that at low frequencies, only the flexural mode significantly couples to the electronic feedback. To validate these assumptions, in Fig. 2 we use COMSOL to compute the lowest 15 vibrational modes for $P > 0$. In panel a, the real part of the first 15 modes are plotted as a function of k, and the modes associated with selected branches are shown in panels b-d. (Modes from every branch are shown Fig. 11.) In panels e-f, we plot the spectrum in the complex plane. Frequencies with negligible imaginary parts are indicated by grey dots, and the remaining frequencies are colored by their wave number k. As can be seen, for frequencies less than $\approx$ 7kHz, only the flexural modes (shown also in Fig. 3 of the main text) have a significant imaginary part. This validates our assumption that that the piezoelectric feedback primarily couples to the flexural modes at low frequencies.

One can ask how the presence of additional vibrational modes affects the computation of the winding number $\nu(\omega)$ in Eq. (30) of the main text. As shown in Fig. 2e, only the flexural mode as a significant imaginary part at low frequencies, and so only flexural modes will be significantly localized in the frequency range of interest. Additionally, one can ask how to reconcile the continuum equation (Eq. 45) with the fact that the shear dominated band contributes to $\nu(\omega)$ despite being an unphysical artifact of the theory. The answer is that the physically relevant piece of information is not necessarily the absolute value of $\nu(\omega)$, but rather the relative value $\nu(\omega') - \nu(\omega'')$ for any two given frequencies ω' and ω''. Suppose, for example, there are n left localized modes at ω' with a given set of boundary conditions. The absolute value of $\nu(\omega')$ and the way in which the boundary conditions are quantified will depend on the details of the theory. However, given only the value of $\nu(\omega') - \nu(\omega'')$, one can conclude that there will be $n - \nu(\omega') + \nu(\omega'')$ left localized modes at ω'' for the same set of boundary conditions. Finally, we note that to compute the relative value $\nu(\omega') - \nu(\omega'')$, one need only draw a line in the complex plane from ω'' to ω' and count the number of signed crossings with the periodic boundary spectrum. Hence, the value $\nu(\omega') - \nu(\omega'')$ only depends on accurately resolving the spectrum in the frequency range of interest, and not on features of the spectrum outside the range of validity of the theory.

H. Simulations of quasistatic energy cycle and finite frequency efficiency

In Fig. 2a-b., we verify Eq. (20) via finite element simulations. We directly compute the work done by a single unit cell of the material that undergoes a path of deformations via fully electromechanically coupled numerical simulations in COMSOL Multiphysics. We enforce displacement boundary conditions on the side boundaries of a metamaterial unit cell. We define the bending curvature $\kappa = \partial_x \varphi$ to be the relative angle of the two terminating cross sections divided by the unit cell's length L, and $\gamma = \partial_x h$ to be the relative vertical displacements of the two cross sections divided by the unit cell's length. In the calculations, geometric and material parameters of the metamaterial are given in Table S1 (See below). We take the beam on a rectangular path through deformation space with $\gamma_{\max} = 9.375 \times 10^{-3}$ and $\kappa_{\max} = 1.0\ m^{-1}$. We subdivide each leg of the path into approximately 150 subdivisions and perform a static analysis at each subdivision to determine the reaction forces F_r and F_l and moments M_l and M_r at the left and right

boundaries, respectively. We then compute the work done on the ith step as:

$$W^i = L\big[(F_r^i - F_l^i)(\gamma^i - \gamma^{i-1}) + (M_r^i - M_l^i)(\kappa^i - \kappa^{i-1})\big] \tag{S48}$$

where the index i labels the step.

For the example shown in the main text, $P = 13.85 \times 10^6\ N/m$ and the volume of the unit cell is $V = 576 \times 10^{-9}\ m^3$. The continuum theory then predicts that the magnitude of the work done should be given by:

$$|W_{\text{theory}}| = P\kappa^{max}\gamma^{max}V = 0.0748\,(J)\,. \tag{S49}$$

In simulation, we find:

$$|W_{\text{sim}}| = \left|\sum_i W^i\right| = 0.0756\,(J)\,, \tag{S50}$$

We note that the continuum equations rely on the approximation of linearity, whereas the COMSOL simulations directly compute the underlying forces and moments based on the microscopic details.

In addition to quasistatic strain controlled deformations, we also examine the efficiency of our metabeam at absorbing energy from finite frequency waves. To do so, we first perform numerical simulations matching the parameters in our experiments. From these numerical tests, we measure the mechanical energy flux from the left and right boundaries of a given unit cell (in this case the fifth unit cell). Denoting these energy fluxes by F_L and F_R, respectively, we define the energy absorption efficiency of left-traveling waves by $\mathcal{E} = (F_R - F_L)/F_R$, where $|F_R| > |F_L|$. At 2 kHz, we find the peak absorption efficiency per unit cell is equal to $\mathcal{E} = 0.38$. Supplementary Figure 6 shows this efficiency at frequencies from 0.6 to 3.0 kHz, where the waves propagating from the right to the left are attenuated.

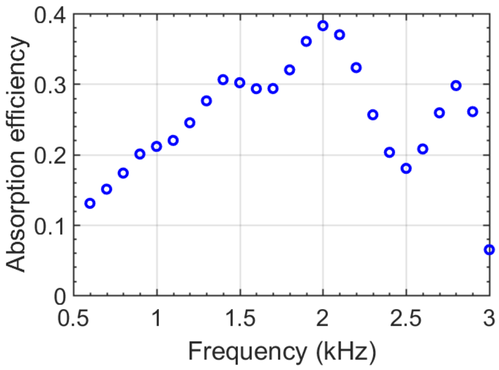

Supplementary Figure 6: The absorption efficiency $\mathcal{E}$ as a function of frequency for an incident flexural wave.

Supplementary Note 2: Experimental details

A. Material parameters

The active metamaterial displayed in main-text Fig. 1 utilizes piezoelectric patches integrated with electronic feedback [5–15]. Supplementary Figure 7 provides a schematic of the odd micropolar metamaterial. Tables S1 and S2 contain the geometric and material parameters, respectively, used in the design.

Supplementary Table 1: Geometric parameters of the odd micropolar metamaterial.

L	32 mm	h_b	3 mm	w_h	6 mm
L_p	6 mm	h_p	0.55 mm	w_p	4 mm
L_1	10 mm	L_2	6 mm		

Supplementary Table 2: Material properties of the odd micropolar metamaterial.

Material properties (Steel)					
E_b	$210.0\ GPa$	G_b	$80.8\ GPa$	ρ_b	$7800.0\ kg\ m^{-3}$
Material properties (PZT 5J)					
s_{11}^E	$16.2 \times 10^{-12}\, m^2 N^{-1}$	d_{33}	$5.93 \times 10^{-10}\, CN^{-1}$		
s_{33}^E	$20.7 \times 10^{-12}\, m^2 N^{-1}$	d_{31}	$-2.74 \times 10^{-10}\, CN^{-1}$		
s_{44}^E	$47 \times 10^{-12}\, m^2 N^{-1}$	d_{15}	$7.41 \times 10^{-10}\, CN^{-1}$		
s_{12}^E	$-4.54 \times 10^{-12}\, m^2 N^{-1}$	ε_{33}^S	$1433.6\, \varepsilon_0$		
s_{13}^E	$-5.9 \times 10^{-12}\, m^2 N^{-1}$	ε_{11}^S	$1704.4\, \varepsilon_0$		
ρ_p	$7700.0\, kg\, m^{-3}$	ε_0	$8.842 \times 10^{-12}\, CmV^{-1}$		

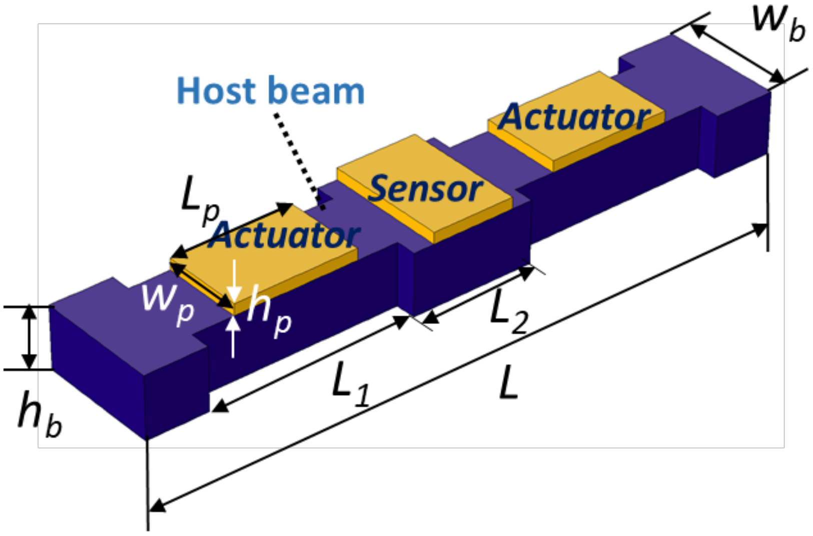

Supplementary Figure 7: Schematic of a unit cell.

B. Electrical control system

Supplementary Figure 8 shows a schematic of the electrical control system used in each unit cell. We use a standard non-inverting voltage amplifier, and all the electrical circuits were fabricated on printed circuit boards (PCBs). The parameters for the electrical components are listed in Table S3. The frequency dependence of the lowpass filter is shown in main text Eq. (40) and depicted in Supplementary Figure 9.

During experiments, we also need to consider stability conditions of the metamaterial. In particular, we find that small feedback effects emerge within individual unit cells associated with imperfections in fabrication. When $|H_0|$ exceeds a critical value, the antisymmetric actuating voltages can no longer produce zero sensing signals in experiments and the system experiences an instability. This critical value is $|H_0| \approx 6$ in our current system, but it is highly dependent on the fabrication details. Due to the self amplification of the beam, the critical value of $|H_0|$ usually decreases as the number of unit cells increases.

Supplementary Table 3: Circuit component parameters.

R1	1 MΩ	R2	9.09 kΩ	R3	15.74 kΩ
C1	1 nF	C2	10 nF	C3	2.2 nF
Op-amp	OPA445				

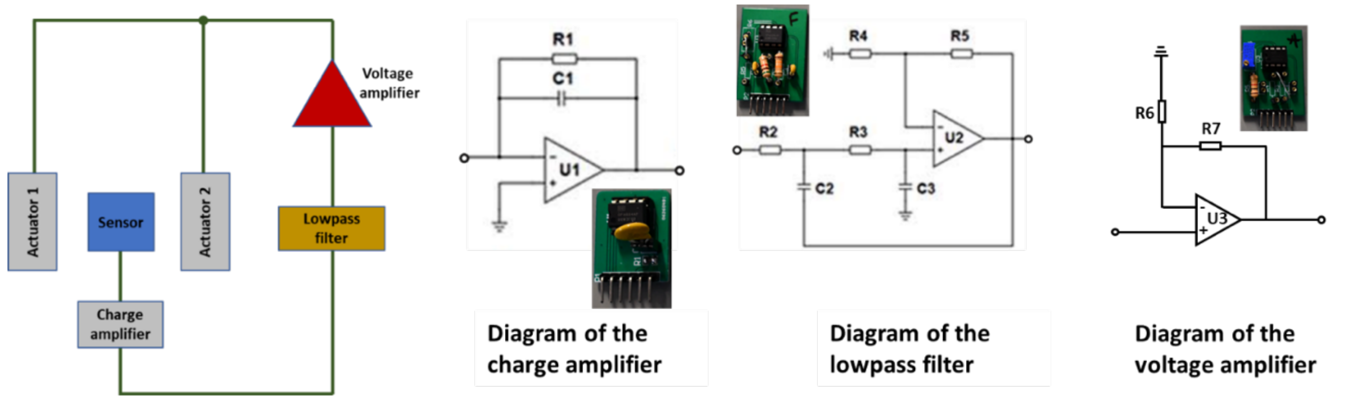

Supplementary Figure 8: The schematic of the electrical control system and circuit diagrams for its individual components.

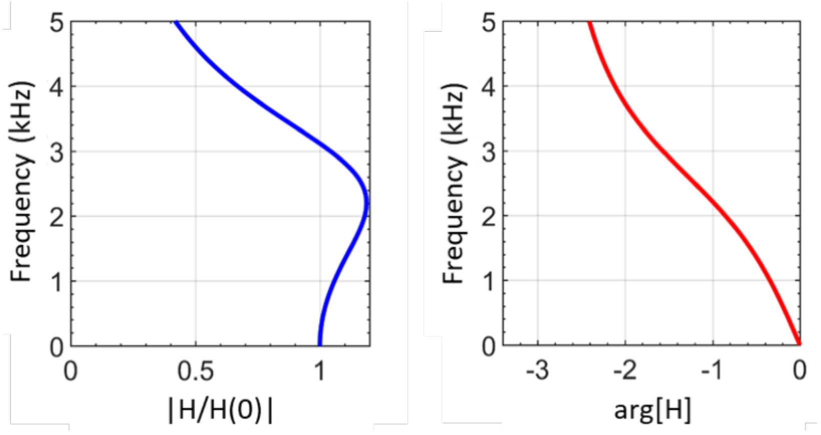

Supplementary Figure 9: Frequency response of the lowpass filter with the transfer function $H(\omega)$. Left: Amplitude; Right: Phase angle.

Supplementary Note 3: Numerics

A. Transfer matrix method for wave dispersion

Here we describe the transfer matrix method [13] used to produce semi-analytical curves in Figures 3 and 6 in the main text. In this approach, the piezoelectric sensing patch is idealized as a point-like strain probe located in the middle each unit cell. This approximation is justified by the large ratio between the experimentally probed wavelengths and the length of the piezoelectric patch (see Supplementary Figure 10). Similarly, piezoelectric actuating patches are idealized as point sources that generate bending moments (see Supplementary Figure 10). With these approximations, the metamaterial unit cell can be divided into seven homogeneous beam sections, shown in Supplementary Figure 10.

For each section, we apply Timoshenko beam assumptions, yielding the following equation of motion for the n-th beam section:

$$B_n \frac{\partial^4 w_n}{\partial x^4} + \left(\frac{B_n \rho_n \omega^2}{\mu_n} + I_n \omega^2 \right) \frac{\partial^2 w_n}{\partial x^2} + \frac{\left(I_n \omega^2 - \mu_n\right) \rho_n \omega^2}{\mu_n} w_n = 0, \tag{S51}$$

where w_n, B_n, ρ_n, ν_n, and I_n denote transverse displacement, bending stiffness, mass density per unit volume, shear modulus and moment of inertia of the n-th homogeneous beam section, respectively. Furthermore, ω is the frequency associated to the Bloch mode $e^{-i\omega t}$. The solutions of w_n read

$$w_n = \bar{A}_n e^{-ik_n x} + \bar{B}_n e^{ik_n x} + \bar{C}_n e^{-\hat{k}_n x} + \bar{D}_n e^{\hat{k}_n x}, \tag{S52}$$

where $k_n = -i\sqrt{\frac{-\alpha_n - \sqrt{\alpha_n^2 - 4B_n\beta_n}}{2B_n}}$ and $\hat{k}_n = \sqrt{\frac{-\alpha_n + \sqrt{\alpha_n^2 - 4B_n\beta_n}}{2B_n}}$ with $\alpha_n = \frac{\rho_n \omega^2 B_n}{\mu_n} + I_n\omega^2$ and $\beta_n = \frac{(I_n\omega^2 - \mu_n)\rho_n\omega^2}{\mu_n}$.

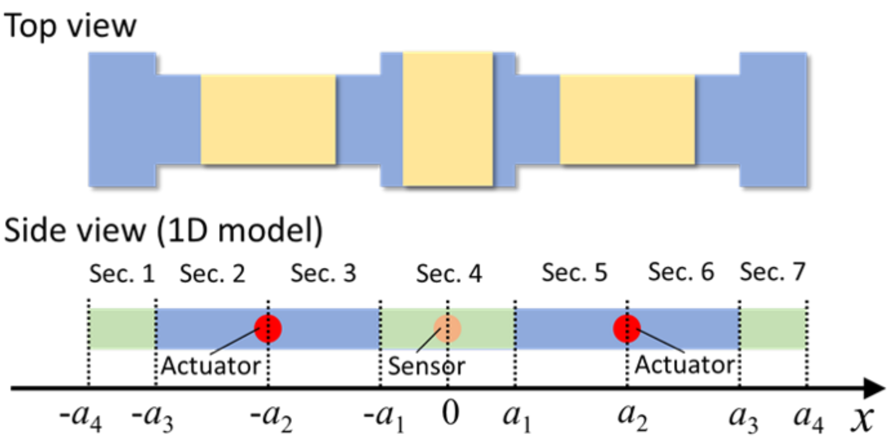

Supplementary Figure 10: Schematic for the transfer matrix method.

In the design, beam sections 1, 4 and 7 possess the same material and geometric parameters. Hence, we may simply write $k_n = k_1$ and $\hat{k}_n = \hat{k}_1$ for these sections. Similarly, we may simply use $k_n = k_2$ and $\hat{k}_n = \hat{k}_2$ for sections 2, 3, 5 and 6. For beam sections 1, 4 and 7, we may summarize the forces and displacement in matrix form by:

$$\mathbf{W}_n(x) = \mathbf{N}_1(x)\,\mathbf{A}_n,\ \ n = 1,4,7 \tag{S53}$$

In Eq. (S53), $\mathbf{W}_n(x) = \begin{bmatrix} w_n(x) & \varphi_n(x) & M_n(x) & F_n(x) \end{bmatrix}^T$, where $\varphi_n(x)$, $M_n(x)$ and $F_n(x)$ are the rotational angle, bending moment and shear force in the n-th beam section. Furthermore, $\mathbf{A}_n = \begin{bmatrix} \bar{A}_n & \bar{B}_n & \bar{C}_n & \bar{D}_n \end{bmatrix}^T$. Lastly,

$$\mathbf{N}_1(x) = \begin{bmatrix} e^{-ik_1x} & e^{ik_1x} & e^{-\hat{k}_1x} & e^{\hat{k}_1x} \\ \frac{-ik_1^2\mu_1+i\omega^2\rho_1}{k_1\mu_1}e^{-ik_1x} & \frac{ik_1^2\mu_1-i\omega^2\rho_1}{k_1\mu_1}e^{ik_1x} & \frac{-\hat{k}_1^2\mu_1-\omega^2\rho_1}{\hat{k}_1\mu_1}e^{-\hat{k}_1x} & \frac{\hat{k}_1^2\mu_1+\omega^2\rho_1}{\hat{k}_1\mu_1}e^{\hat{k}_1x} \\ \frac{-k_1^2\mu_1+\omega^2\rho_1}{\mu_1}B_1e^{-ik_1x} & \frac{-k_1^2\mu_1+\omega^2\rho_1}{\mu_1}B_1e^{ik_1x} & \frac{\hat{k}_1^2\mu_1+\omega^2\rho_1}{\mu_1}B_1e^{-\hat{k}_1x} & \frac{\hat{k}_1^2\mu_1+\omega^2\rho_1}{\mu_1}B_1e^{\hat{k}_1x} \\ \frac{-i\omega^2\rho_1S_1}{k_1}e^{-ik_1x} & \frac{i\omega^2\rho_1S_1}{k_1}e^{ik_1x} & \frac{\omega^2\rho_1S_1}{\hat{k}_1}e^{-\hat{k}_1x} & \frac{-\omega^2\rho_1S_1}{\hat{k}_1}e^{\hat{k}_1x} \end{bmatrix}$$

where S_n is the area of the cross-section of the n-th beam. Similarly, for beam sections 2, 3, 5 and 6, Eq. (S53) reads

$$\mathbf{W}_n(x) = \mathbf{N}_2(x)\,\mathbf{A}_n,\ \ n = 2,3,5,6 \tag{S54}$$

where $N_2(x)$ is obtained by replacing the index "1" in $N_1(x)$ by "2".

The effective point source vector generated by the left actuator can be written as

$$\mathbf{G} = \mathbf{H}\mathbf{A}_4 \tag{S55}$$

where $\mathbf{G} = \begin{bmatrix} 0 & 0 & M_a & 0 \end{bmatrix}^T$ with M_a being the effective bending moment produced by the actuator, and

$$\mathbf{H} = \begin{bmatrix} 0 & 0 & 0 & 0 \\ 0 & 0 & 0 & 0 \\ H\kappa_a\kappa_s & H\kappa_a\kappa_s & H\kappa_a\kappa_s & H\kappa_a\kappa_s \\ 0 & 0 & 0 & 0 \end{bmatrix}$$

where κ_a and κ_s denote electromechanical coupling coefficients of the piezoelectric actuator and sensor, respectively, which are retrieved from finite-element simulations.

Next, we impose continuity conditions on the transverse displacement, rotational angle, bending moment and shear force at the section boundaries ($x = -a_3, -a_2, -a_1, a_1, a_2$ and a_3) to obtain:

$$\begin{aligned} &\mathbf{N}_1(-a_3)\,\mathbf{A}_1 = \mathbf{N}_2(-a_3)\,\mathbf{A}_2, \\ &\mathbf{N}_2(-a_2)\,\mathbf{A}_2 = \mathbf{N}_2(-a_2)\,\mathbf{A}_3 + \mathbf{H}\mathbf{A}_4, \\ &\mathbf{N}_2(-a_1)\,\mathbf{A}_3 = \mathbf{N}_1(-a_1)\,\mathbf{A}_4, \\ &\mathbf{N}_1(a_1)\,\mathbf{A}_4 = \mathbf{N}_2(a_1)\,\mathbf{A}_5, \\ &\mathbf{N}_2(a_2)\,\mathbf{A}_5 = \mathbf{N}_2(a_2)\,\mathbf{A}_6 - \mathbf{H}\mathbf{A}_4, \\ &\mathbf{N}_2(a_3)\,\mathbf{A}_6 = \mathbf{N}_1(a_3)\,\mathbf{A}_7. \end{aligned} \tag{S56}$$

Eq. (S56) can be expressed as

$$\mathbf{A}_7 = \mathbf{T}\mathbf{A}_1, \tag{S57}$$

where the transfer matrix $\mathbf{T}$ is given by

$$\begin{aligned}\mathbf{T} = \mathbf{N}_1^{-1}(a_3)\,\mathbf{N}_2(a_3)\,\mathbf{N}_2^{-1}(a_2)\left[\mathbf{N}_2(a_2) + \mathbf{H}\mathbf{N}_1^{-1}(a_1)\,\mathbf{N}_2(a_1)\right]\mathbf{N}_2^{-1}(a_1)\,\mathbf{N}_1(a_1)\,\mathbf{N}_1^{-1}(-a_1)\times \\ \mathbf{N}_2(-a_1)\left[\mathbf{N}_2(-a_2) + \mathbf{H}\mathbf{N}_1^{-1}(-a_1)\,\mathbf{N}_2(-a_1)\right]^{-1}\mathbf{N}_2(-a_2)\,\mathbf{N}_2^{-1}(-a_3)\,\mathbf{N}_1(-a_3)\,.\end{aligned} \tag{S58}$$

Applying Bloch theorem on the left and right edges of the unit cell gives

$$\mathbf{N}_1(-a_4)\,\mathbf{A}_1 = e^{ikL}\mathbf{N}_1(a_4)\,\mathbf{A}_7. \tag{S59}$$

Combining Eqs. (S57) and (S59), one can derive

$$e^{ikL}\left[\mathbf{N}_1^{-1}(-a_4)\,\mathbf{N}_1(a_4)\,\mathbf{T}\right]\mathbf{A}_1 = \mathbf{A}_1. \tag{S60}$$

Solving the eigenvalue problem in Eq. (S60) for imposed frequencies, one can obtain the corresponding complex wavenumbers.

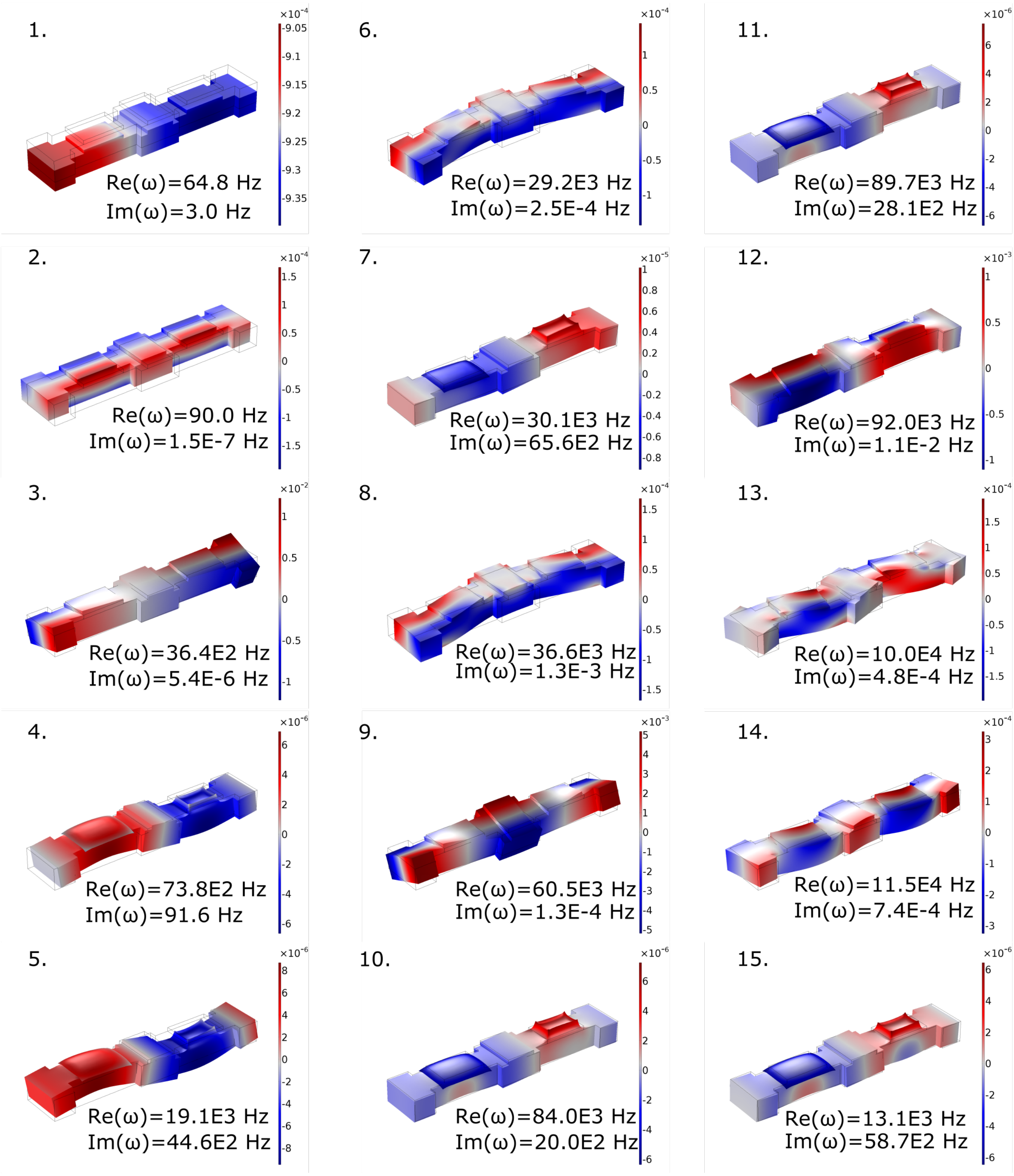

Supplementary Figure 11: The lowest 16 modes corresponding to bands shown in Fig. 2. The wavenumber is taken to be $kL = 0.314$, where L is the lattice spacing. The odd micropolar modulus is $P = 3\Pi$. The color bar denotes the z (vertical) component of the displacement field.

Supplementary References

[1] Timoshenko, S. *Elements of strength of materials* (Van Nostrand, Princeton, N. J., 1949), 3d ed. edn.

[2] Brandenbourger, M., Locsin, X., Lerner, E. & Coulais, C. Non-reciprocal robotic metamaterials. *Nature Communications* **10**, 4608 (2019).

[3] Trefethen, L. & Embree, M. *Spectra and Pseudospectra: The Behavior of Nonnormal Matrices and Operators* (Princeton University Press, 2020).

[4] Lee, C. H. & Thomale, R. Anatomy of skin modes and topology in non-hermitian systems. *Phys. Rev. B* **99**, 201103 (2019).

[5] Chen, Y. Y., Huang, G. L. & Sun, C. T. Band Gap Control in an Active Elastic Metamaterial With Negative Capacitance Piezoelectric Shunting. *Journal of Vibration and Acoustics* **136** (2014). 061008.

[6] Bergamini, A. *et al.* Phononic crystal with adaptive connectivity. *Advanced Materials* **26**, 1343–1347 (2014).

[7] Chen, Y. Y., Zhu, R., Barnhart, M. V. & Huang, G. L. Enhanced flexural wave sensing by adaptive gradient-index metamaterials. *Scientific Reports* **6**, 35048 (2016).

[8] Yi, K., Collet, M., Chesne, S. & Monteil, M. Enhancement of elastic wave energy harvesting using adaptive piezo-lens. *Mechanical Systems and Signal Processing* **93**, 255 – 266 (2017).

[9] Alan, S., Allam, A. & Erturk, A. Programmable mode conversion and bandgap formation for surface acoustic waves using piezoelectric metamaterials. *Applied Physics Letters* **115**, 093502 (2019).

[10] Chen, Y., Hu, G. & Huang, G. A hybrid elastic metamaterial with negative mass density and tunable bending stiffness. *Journal of the Mechanics and Physics of Solids* **105**, 179 – 198 (2017).

[11] Wang, G., Cheng, J., Chen, J. & He, Y. Multi-resonant piezoelectric shunting induced by digital controllers for subwavelength elastic wave attenuation in smart metamaterial. *Smart Materials and Structures* **26**, 025031 (2017).

[12] Casadei, F., Delpero, T., Bergamini, A., Ermanni, P. & Ruzzene, M. Piezoelectric resonator arrays for tunable acoustic waveguides and metamaterials. *Journal of Applied Physics* **112**, 064902 (2012).

[13] Chen, Y. Y., Hu, G. K. & Huang, G. L. An adaptive metamaterial beam with hybrid shunting circuits for extremely broadband control of flexural waves. *Smart Materials and Structures* **25**, 105036 (2016).

[14] Sugino, C., Ruzzene, M. & Erturk, A. Merging mechanical and electromechanical bandgaps in locally resonant metamaterials and metastructures. *Journal of the Mechanics and Physics of Solids* **116**, 323 – 333 (2018).

[15] Lakes, R. Giant enhancement in effective piezoelectric sensitivity by pyroelectric coupling. *EPL (Europhysics Letters)* **98**, 47001 (2012).
