# Supplementary material for: Realization of active metamaterials with odd micropolar elasticity
Source: Nat Commun. 2021 Oct 12;12:5935. doi: 10.1038/s41467-021-26034-z (PMC8511045; doi:10.1038/s41467-021-26034-z)
Supplement: Supplementary file 3 — Description of Additional Supplementary Files [file 41467_2021_26034_MOESM3_ESM.pdf]

Description of Additional Supplementary Files

File name: Supplementary Movie 1
Description: Unidirectional amplification. Experimentally measured transverse velocity wave field in response to excitation on the left. The incident wave is a tone burst signal centred at 2 kHz.

File name: Supplementary Movie 2
Description: Unidirectional attenuation. Experimentally measured transverse velocity wave field in response to excitation on the right. The incident wave is a tone burst signal centered at 2 kHz.
